# Nanoparticles for Interrogation of Cell Signaling

**DOI:** 10.1146/annurev-anchem-092822-085852

**Published:** 2023-06-14

**Authors:** Seonik Lee, Mengchi Jiao, Zihan Zhang, Yan Yu

**Affiliations:** Department of Chemistry, Indiana University, Bloomington, Indiana, USA

**Keywords:** nanoparticles, cell signaling, intracellular sensing, magnetic manipulation, optical manipulation, quantitative imaging

## Abstract

Cell functions rely on signal transduction—the cascades of molecular interactions and biochemical reactions that relay extracellular signals to the cell interior. Dissecting principles governing the signal transduction process is critical for the fundamental understanding of cell physiology and the development of biomedical interventions. The complexity of cell signaling is, however, beyond what is accessible by conventional biochemistry assays. Thanks to their unique physical and chemical properties, nanoparticles (NPs) have been increasingly used for the quantitative measurement and manipulation of cell signaling. Even though research in this area is still in its infancy, it has the potential to yield new, paradigm-shifting knowledge of cell biology and lead to biomedical innovations. To highlight this importance, we summarize in this review studies that pioneered the development and application of NPs for cell signaling, from quantitative measurements of signaling molecules to spatiotemporal manipulation of cell signal transduction.

## 1. INTRODUCTION

Cells sense their extracellular environment through individual receptor-ligand recognition at the plasma membrane. Information from the initial receptor activation is relayed from the cell surface to the nucleus through the molecular interactions and biochemical reactions of the signal transduction cascades. This complex process of information transfer is also known as signal transduction. The signal from a single receptor often diverges and then converges again through multiple signaling pathways. Receptors and their signaling pathways often work in combination to recognize complex extracellular stimuli, such as the presence of a particular pathogen. The molecular interactions and enzyme reactions—when they occur, where they occur, and how they fluctuate with time—govern the cell response outcome (1-4). Mechanisms by which cell signaling processes are orchestrated dictate how cells function and how they respond to therapeutic interventions (5, 6).

A better understanding of cell signaling mechanisms is key to generating new insights into basic cell biology and new biomedical innovations. The complexity of processes such as signal transduction in the cell is a major barrier to this understanding. Traditional biochemical and cell biology tools have made it possible to identify the elements of cell signaling pathways, but such methods often lack the high spatiotemporal resolution needed to characterize such highly dynamic cellular processes (7). In recent years, nanoparticle (NP)-based toolsets have emerged as a major new strategy for overcoming the barriers to understanding and manipulating these processes (8-11). NPs have unique optical, magnetic, chemical, and physical properties that distinguish them from bulk materials (12, 13). Importantly, these unique material properties can be controlled by manipulating accessible parameters such as the size, shape, surface functionality, and chemical composition of NPs. This unique versatility has enabled new studies in which cell signaling can be visualized, quantified, and controlled in real time with high spatiotemporal precision. There are many examples. These range from NP-based optical sensors for quantifying intracellular biochemical signals (14-16) to NP actuators that can harness optical and magnetic forces to remotely control cell signaling, both on the cell surface and inside the cell (17-20). In this review, we summarize those pioneering studies. Our goal is to draw attention to the remarkable potential of NP-enabled bio-analytical and biophysical tools for the quantitative understanding of cell functions—an area of research that is still in its infancy but can potentially transform our knowledge of cell biology. We note that the use of NPs for the intracellular delivery of genetic material (21-23) is also, technically, a way of manipulating cell signaling, but those studies are focused on therapeutic applications rather than fundamental research and are therefore outside our focus of interest.

## 2. OPTICAL NANOPARTICLE PROBES FOR INTRACELLULAR SENSING OF SIGNALING MOLECULES

Intracellular signaling and other biochemical activities fluctuate with time and subcellular location. Real-time imaging of some optical detectors is the most direct way to capture these spatiotemporal dynamics. Significant progress toward this goal has been made using analyte-sensitive fluorescent probes. But small-molecule probes share some major limitations. They must be capable of entering a cell by crossing its membrane and becoming membrane-impermeable once inside, which is challenging to achieve. They are often nonspecifically internalized and sequestered by cells, leading to high background noise for optical imaging. They often have relatively poor detection sensitivity because of photoinstability and low brightness. Many of these limitations can be overcome by using NP-based intracellular sensors. NP optical probes can pack many of the small-molecule probes into a single entity. This provides sufficient brightness and the photostability needed for observations on long timescales. Some NP probes enable detection of analytes by mechanisms different from those of small-molecule probes. NP probes can also be delivered into cells by various methods. Unlike small molecules, they can be engulfed by cells via the natural process of endocytosis or injected into cells ballistically. Such methods can enable analyte sensing in specific subcellular locations.

### 2.1. Intracellular Proton Fluxes

Proton fluxes across the plasma membrane and intracellular membranes are tightly regulated to maintain intracellular pH and regulate the cotransport of nutrients, metabolites, and other types of ions (24-27). Spatial and temporal fluctuations of intracellular proton concentrations play a direct role in cell growth, proliferation, and apoptosis (25, 28). Various types of pH-sensitive NP probes have been developed to monitor and quantify intracellular proton fluxes inside living cells. The typical design is to incorporate a large number of pH-sensitive fluorophore indicators into a single NP. This can be achieved by physical encapsulation or covalent conjugation. The pH indicators, which are often fluorescent dyes, change their fluorescence properties when they bind to protons. One potential concern, however, is that changes in the fluorescence intensity of a single pH probe do not necessarily indicate pH change, because it can also be affected by other factors, such as the intracellular movement of NP probes along the direction perpendicular to the focal plane of the microscope objective. This problem can be overcome by comparing the ratio of two different fluorescences through ratiometric imaging rather than measuring the absolute brightness of a single fluorophore. To achieve this, the NP probe must contain a secondary fluorophore that is either insensitive to pH or has a pH sensitivity opposite of that of the pH indicator. For example, Wu et al. (29) monitored lysosomal pH in living cells by using mesoporous silica NPs functionalized with both rhodamine-lactam and fluorescein isothiocyanate (FITC). Rhodamine-lactam fluorescence increases with decreasing pH, whereas FITC fluorescence responds oppositely. This response opposition enabled ratiometric imaging of lysosomes to reliably detect a broad pH range of 6.0–3.5. By using triple-labeled NP sensors in endosomes, Benjaminsen et al. (30) demonstrated that the range of pH detection can be broadened to 7.0–3.2 with improved detection accuracy.

**Figure 1:**
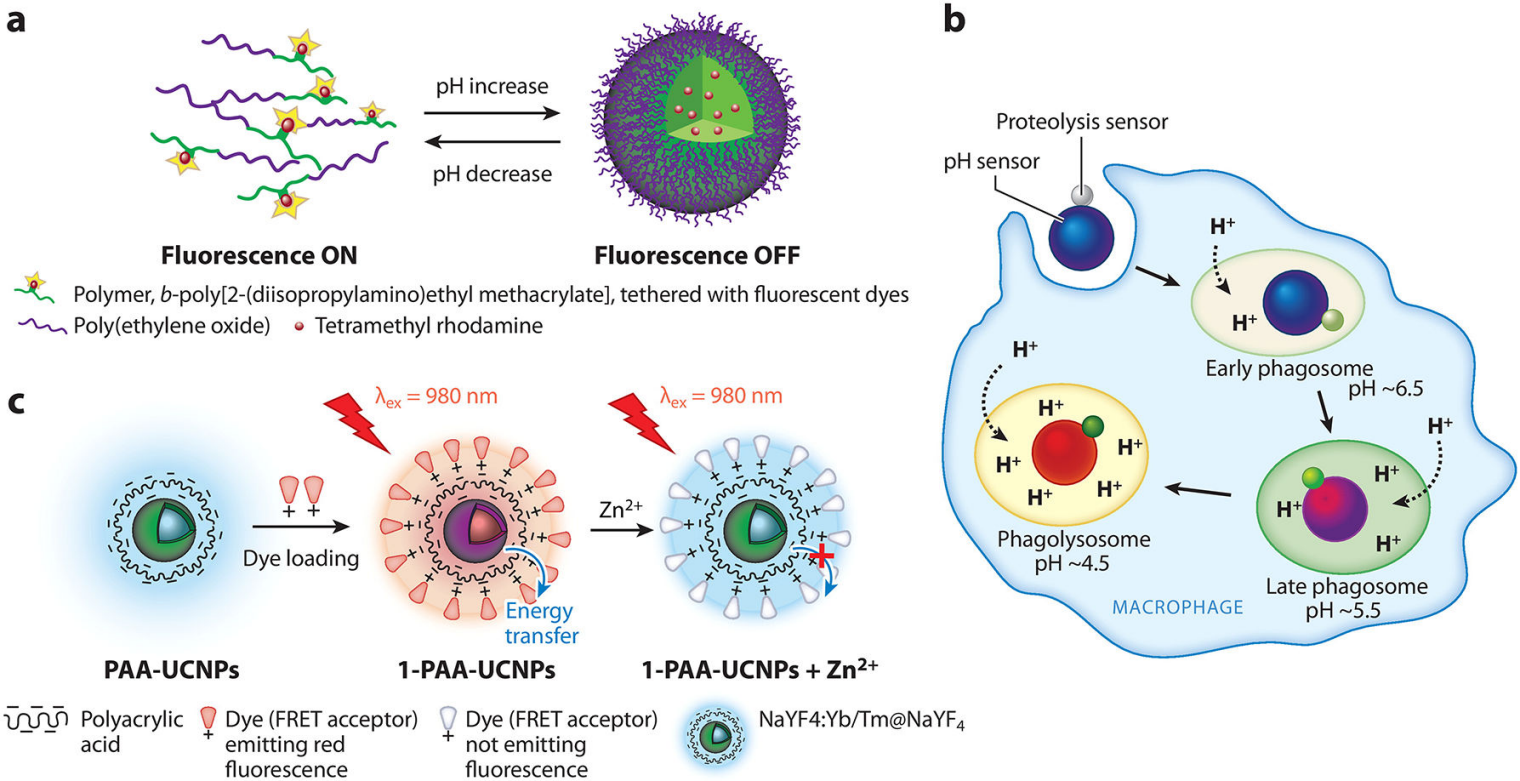
Examples of NP optical probes for intracellular sensing of signaling molecules. (*a*) Schematic illustration of the design of pH-sensing NP probes based on pH-induced micellization of polymers, leading to fluorescence switching between the ON (unimer) and OFF (micelle) states. Panel adapted with permission from Reference 31; copyright 2012 American Chemical Society. (*b*) Schematic illustration of bifunctional Janus particle probe that contains spatially separated modules for pH and proteolysis sensing. The probe was used for measuring phagosome maturation from early to late phagosomes and then to phagolysosomes. The pH-sensing module emits strong fluorescence in an acidic pH environment (as indicated by the blue-to-red color change of the large particle). The proteolysis-sensing module releases fluorogenic peptides (as indicated by the grey-to-green color change of the small particle) upon activation of proteolytic enzymes in phagosomes. Color change of the phagosome indicates the remodeling of its membrane and lumen during maturation. Panel adapted with permission from Reference 34; copyright 2021 John Wiley & Sons. (*c*) Schematic illustration of the synthesis of chromophore-containing UCNPs and their luminescence changes in response to Zn^2+^. The UCNPs emit blue fluorescence by themselves, but they emit red fluorescence after the conjugation of dyes on the surface due to a FRET process. The presence of Zn^2+^ disrupts the FRET process, resulting in blue fluorescence emission from UCNPs. Panel adapted with permission from Reference 52; copyright 2015 American Chemical Society. Abbreviations: FRET, Förster resonance energy transfer; NP, nanoparticle; PAA, polyacrylic acid; UCNP, upconversion nanoparticle.

Because most small-molecule pH indicators detect protons indirectly via their ionizable residues, their fluorescence is not sensitive to subtle pH changes. NP probes that employ small-molecule pH indicators share this same limitation. To make NP probes with a higher sensitivity to small pH changes, Zhou et al. (31) developed NP probes that change fluorescence emission with pH based on pH-induced micellization of polymers (Figure 1a). The polymers are tethered to fluorophores that have a small Stokes shift (Δλ < 40 nm) so they can have homo Förster resonance energy transfer (homoFRET). When the polymers form micelles at a given pH, the homoFRET between the fluorophores causes ratiometric spectral changes. Those NP probes are reportedly sensitive for pH changes as small as 0.2 pH units. Furthermore, the requisite polymer chemistry can be modified to optimize detection sensitivity for different pH conditions.

To understand possible correlations between proton fluxes and the spatiotemporal distribution of other signaling molecules inside the cell, dual-functional NP optical probes have been developed for simultaneous imaging. A key technical challenge is to minimize the optical interference between the two detection modules. One strategy is to create spatially separated compartments in a single NP probe. Wang et al. (32) reported the development of an NP probe for the simultaneous measurement of pH and oxygen concentrations in the cell cytosol. The NP probe has a core-shell polymer structure, in which the core matrix contains an oxygen indicator, while the polymer shell was conjugated with a pH indicator. As a proof of concept, the authors showed that the dual NP probes fluoresce strongly after delivery into cells via electroporation, but they provided little quantification of pH and oxygen concentration in cells. A similar core-shell NP design was used by Pan et al. (33) to demonstrate the simultaneous monitoring of pH and dissolved oxygen in macrophage cells. Recently, our group (34) reported a Janus particle probe that contains two covalently linked compartments: one for pH sensing and the other for proteolysis (Figure 1b). Using this particle probe, we identified the threshold acidic pH inside phagosomes needed to initiate and maintain proteolysis for efficient pathogen degradation. Overall, this emerging area has so far focused mostly on the synthesis of NP probes, but the biological insights obtained from these probes have, to date, been limited.

Quantum dots (QDs) represent an appealing alternative to dye-conjugated NPs for intracellular pH sensing because their optical properties are highly sensitive to charge transfer. QDs, with appropriate surface ligands, can function as charge-transfer-coupled pH sensors (35, 36). For example, Medintz et al. (36) developed QD–dopamine–peptide conjugates for intracellular pH imaging. Dopamine hydroquinone serves as the electron acceptor and a quencher for the QD. Its oxidation state changes with pH, causing the fluorescence intensity of QDs to vary. The fluorescence lifetime of the QDs also changes, providing an alternative and possibly a more sensitive pH indicator (37). In addition to QDs, carbon nanodots and upconversion NPs are emerging new materials for intracellular pH sensing (38, 39). Upconversion NPs exploit the quantum phenomenon of photon upconversion, in which the absorption of two lower-energy photons causes the emission of a single higher energy photon. Both types of NPs have intrinsic pH-sensitive photoluminescence and are more photostable than dye-conjugated NP probes.

### 2.2. Intracellular Metal Ions

Metal ions, such as Zn^+^ and Cu^2+^, are indispensable cofactors in many cell signaling pathways (40-42). Ca^2+^, for example, regulates muscle contraction and cellular metabolism (43), and Mg^2+^ is a prerequisite for protein and nucleic acid synthesis (44). Monitoring the spatiotemporal distribution and fluctuation of the concentrations of those metal ions in cells can yield critical insights into how signal transduction is regulated. Optical imaging of the concentration of metal ions in living cells has been enabled by the development of small-molecule fluorescent indicators sensitive to metal ions. However, such indicators suffer from limitations, including low brightness and high cytotoxicity. These limitations can be overcome by incorporating small-molecule metal ion indicators into NPs for live cell imaging. Kopelman and coworkers (45-47) developed the so-called nano-PEBBLE sensors, in which metal ion indicators for Ca^2+^, Mg^2+^, Zn^2+^, or Fe^3+^ are encapsulated inside porous hydrogel NPs. The encapsulation of small-molecule indicators in a porous matrix reduces their cytotoxicity by preventing them from diffusing freely in cells, yet still allows them to be accessible to analytes. The surfaces of nano-PEBBLE sensors can be further modified with ligands that target specific organelles in the cell. Ding et al. (48), for example, reported a ratiometric NP sensor designed to detect Cl^−^ concentration in lysosomes. The outer surfaces of these NPs were conjugated with a Cl^−^-sensitive dye, whereas the matrix was embedded with a Cl^−^-insensitive reference dye. This spatial separation between the dyes minimized their potential optical interference. The fluorescence lifetime of both the indicator and reference dye provides a sensitive indicator of Cl^−^ concentration in the range of 0–200 mM inside lysosomes.

The FRET between different fluorophores can also be exploited as an indicator for metal ion detection (49). For example, Zhu et al. (50) developed a gold NP sensor that uses the FRET signal as an indicator for detecting Pb^2+^ in lysosomes. Gold NPs were conjugated with Pb^2+^-specific deoxyribozymes (DNAzymes). DNAzymes are oligonucleotides containing sequences that can facilitate specific chemical reactions, such as binding to a specific ion such as Pb^2+^. DNA strands that are substrate to the DNAzyme are labeled with a fluorophore on one end and a quencher on the other end that quenches the fluorescence. When the DNAzymes bind to Pb^2+^, they cleave the substrate DNA into short strands. This cleaving increases the distance between the fluorophore and the quencher and therefore results in an increase in fluorescence. An advantage of this design is that the DNAzymes can be modified to capture different metal ions. However, the temporal resolution of this type of NP sensor is limited because of the multiple chemical steps of binding and cleavage required. The poor temporal resolution of metal ion detection is obvious in many studies, in which the interval of measurements is typically tens of minutes or longer (51). Peng et al. (52) demonstrated significantly improved temporal resolution in the detection of Zn^2+^ in cells, with <1-μM sensitivity and 5-s temporal resolution. In this study, upconversion luminescence was quenched by chromophores coated on the NP surface, with the quenching reversed once the chromophore absorption blue shifts in the presence of Zn^2+^ (Figure 1c).

### 2.3. Intracellular Oxygen

Molecular oxygen is another substance whose concentration within cells is of special interest. Oxygen’s role in the cell is double sided. On the one hand, its presence is critical to oxidative respiration, by which cells generate energy, but on the other hand, it serves as a substrate in enzymatic reactions that generate oxidative damage to the cell (53, 54). Intracellular oxygen concentration also appears to play a role in tumor progression. Tumor cells are known to have a lower level of intracellular oxygen than normal cells (55). Optical imaging of intracellular oxygen concentrations has been enabled primarily by the synthesis of oxygen-sensitive phosphorescent probes, such as Pt(II)-porphyrin derivatives (56), Ru(II) complexes (57), and Ir(III) complexes (58). Those small-molecule probes suffer from several limitations, including poor photostability, insufficient brightness, toxic effect on cell metabolism, nonspecific binding to proteins, and sometimes minimal uptake into cells. Most of those problems can be mitigated by embedding the small-molecule probes in the matrices of NPs. Fercher et al. (59) synthesized NPs in which oxygen-sensitive Pt(II)-porphyrin derivatives were embedded in a cationic polymer matrix. After the NPs are internalized into endosomes, the phosphorescence lifetime of the Pt(II)-porphyrins was found to change as the oxygen concentrations in endosomes changed in response to extracellular conditions (59). Similarly, Coogan et al. (60) have used NPs with embedded Ru(II) complexes for measuring oxygen concentrations in yeast and human cell lines. Together with oxygen-sensitive probes, an oxygen-insensitive reference dye can also be embedded in the NP matrix to achieve ratiometric imaging for improved accuracy of detection (61). In addition, targeting ligands can be coated onto the surface of NPs to measure intracellular oxygen levels in a specific cell or organelle type (61). For example, by coating oxygen-sensitive NP probes with ligands that target mitochondria, Wang et al. (62) measured mitochondrial oxygen consumption rates in cells by using time-resolved luminescence as an indicator. They revealed that mitochondria in tumor cells are distinctly less active than those in healthy cells. This study demonstrated the feasibility of site-specific subcellular sensing of oxygen in living cells.

### 2.4. Intracellular Temperature

Temperature directly impacts all biochemical reactions in living cells. Therefore, measuring the fluctuation and distribution of temperature within the cell is important for understanding its role, and that of thermoregulatory mechanisms, in many cellular events. This includes the pathogenesis of diseases (63). Small-molecule luminescence thermometers have been developed for intracellular temperature sensing through imaging. Besides small-molecule thermometers, nanothermometers have been developed based on polymers (64), rare-earth-doped NPs (65), metal NPs (66), semiconductor nanocrystals (67), carbon dots (68), and other materials (69). Okabe et al. (70) reported the first example of a nanothermometer for subcellular temperature mapping in living cells. In their thermosensitive fluorescent polymeric probe, the block copolymer backbone contained a thermo-responsive poly(*N*-*n*-propylacrylamide) sequence that switches between a collapsed and an extended conformation upon heating or cooling, respectively. As the polymer backbone undergoes conformational change, it changes the hydration environment surrounding the water-sensitive fluorophores that are tethered on the polymer backbone, leading to changes in the fluorescence lifetime and intensity of the fluorophores. Using this method, temperature changes in various intracellular organelles in different cell types were measured within the range of 20–50°C. This is an example of a nanothermometer design in which the temperature-sensing module and luminescence temperature indicator are separated. However, not all nanothermometers have this design. For NPs doped with rare-earth ions and transition-metal ions, the temperature-sensing module is also the temperature indicator. Because the luminescence of those ions is thermosensitive (71, 72), temperature is measured more directly. A variety of NPs doped with rare-earth or transition-metal ions have been developed for ratiometric temperature measurements, such as Ln^3+^-containing polymeric micelles (73), europium (Eu^3+^)-doped silicon NPs (74), and lipoic acid-protected gold nanoclusters (75). Notably, the study by Pinol et al. (73) revealed a heterogeneous distribution of intracellular temperature. The doped NPs used in their study were sensitive for a broad range of temperatures and more photostable than fluorophore-based sensors.

Rare-earth-doped NPs that utilize upconversion are an emerging type of NPs that exhibit superior photostability and brightness. NP thermometers based on ratiometric upconversion have been developed to monitor the thermal dynamics in mitochondria in living cells (76). Qiu et al. (77) developed a ratiometric upconversion NP thermometer that has dual emission at the same excitation wavelength. The nanothermometer is a hybrid upconversion nanocluster that contains a mixture of upconversion-emissive PbS QDs that are thermosensitive and Tm-doped upconversion NPs as the reference. The near-infrared (NIR) excitation and emission of the hybrid nanomaterial are advantageous for penetrating tissues during in vivo imaging. As a proof of concept, the nanothermometers were used to monitor temperatures inside a tumor in mice during photothermal therapy.

## 3. NANOPARTICLES FOR SIGNALING MANIPULATION FROM THE CELL SURFACE

### 3.1. Manipulation of Ion Channel Activities

**Figure 2:**
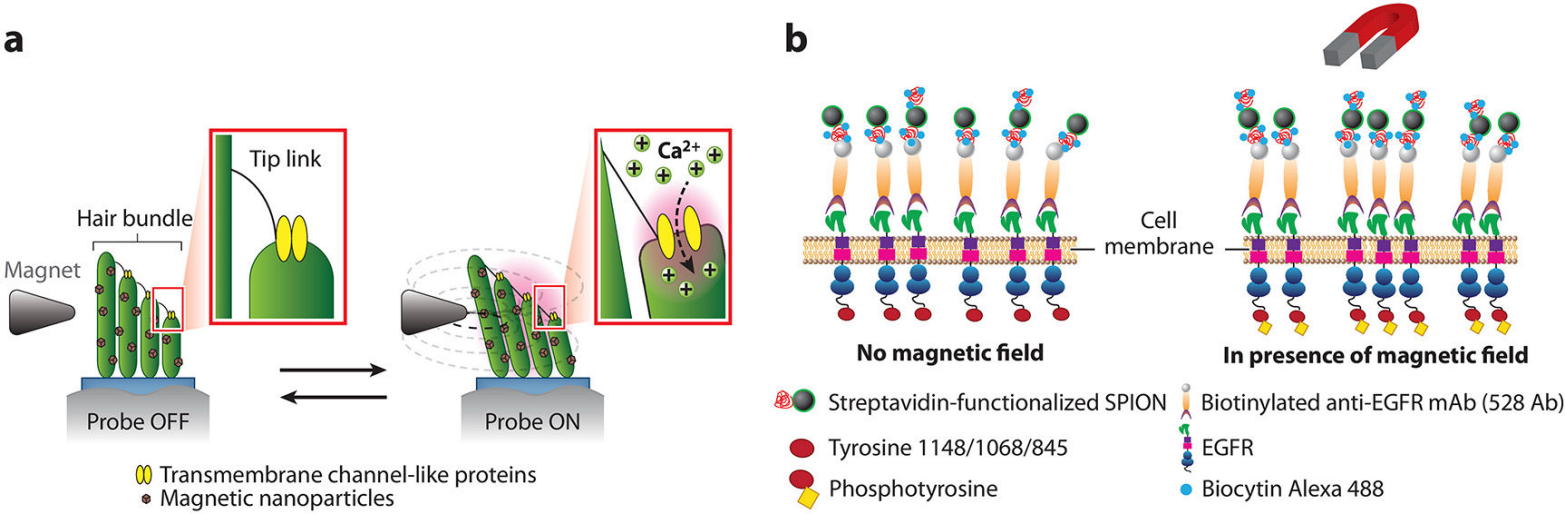
Examples of signaling manipulation from the cell surface using magnetic NPs. (*a*) Schematic illustration of magnetic control of the opening and closing of Ca^2+^ channels on inner ear hair cells. Magnetic manipulation induces mechanical tension in tip links that tether the stereocilia of hair cells with the extracellular environment. The mechanical tension activates ion channels for Ca^2+^ influx. Panel adapted with permission from Reference 80; copyright 2014 American Chemical Society. (*b*) Schematic illustration of using magnetic NPs to activate EGFRs in cell plasma membrane. The activation occurs without ligand-receptor recognition, as the EGFR binding pockets were blocked with antibodies. Panel adapted from Reference 107 (CC BY 4.0). Abbreviations: EGFR, epidermal growth factor receptor; mAb, monoclonal antibody; NP, nanoparticle; SPION, superparamagnetic iron oxide NP.

Mechanosensitive ion channels are a ubiquitous feature of all cell membranes. In response to mechanical stimuli, these channels change their conformation to generate ion flux signals (78). When a magnetic NP is tethered to a mechanosensitive ion channel on the plasma membrane of a cell, an applied magnetic force can then be used to mechanically activate the channel. There are two major advantages of this approach to studying the functions of these channels. First, magnetic fields readily penetrate deep tissue without causing cell damage and, second, magnetic force allows mechanical manipulation of a channel with consistency and precision (79). Unfortunately, however, a conventional magnetic tweezers setup often cannot generate magnetic forces with a sufficient spatial and temporal resolution to control the opening and closing of ion channels with NPs for quantitative measurements. To overcome this limitation, some have tried to modify the material properties of magnetic NPs. For example, Lee et al. (80) modified the NP shape. They used cube-shaped NPs, rather than spherical magnetic NPs, to apply piconewton-scale forces to the stereocilia of inner ear hair cells (Figure 2a). Compared to spherical magnetic NPs, magnetic nanocubes were found to enable ultrafast (submilliseconds) mechanical control with high spatial precision (displacement of tens of nanometers). Alternatively, Tay et al. (81) created microfabricated magnetic substrates, which, used in conjunction with magnets, generate magnetic field gradients that are highly localized and have orders of magnitude greater strength than what would typically be expected by using magnets alone. This allowed the remote control of N-type mechanosensitive calcium ion channels for neural cell stimulation via mechanical stretching of the plasma membrane (82). A crucial limitation in using conventional magnetic tweezers for manipulating magnetic NPs is that the magnetic tip must be positioned within micrometer proximity to the magnetic NPs to generate sufficiently large force. For this reason, this sort of magnetic manipulation cannot be used for in vivo manipulation in deep tissue samples or in live animals. To overcome this limitation, Lee et al. (79) developed so-called m-Torquer magnetic NPs, each of which is a 500-nm spherical polystyrene bead covered with a dense layer of 25-nm octahedral ferromagnetic NPs. Owing to their magnetic anisotropy, the m-Torquer magnetic NPs generate piconewton torque force under a rotating magnetic field. This force was sufficient to successfully stimulate the mechanosensitive Piezo1 channels in cells and also in the brains of freely moving mice.

Besides mechanosensitive ion channels, cells also express a variety of thermosensitive ion channels involved in thermal regulation. To control the activation of those ion channels, localized heat is typically generated by using magnetic or plasmonic NPs. Under an alternating magnetic field, superparamagnetic NPs can convert electromagnetic energy to heat via three independent mechanisms: Néel relaxation, Brownian relaxation, and hysteresis loss (83). Once magnetic NPs bind to the specific thermosensitive ion channels, their localized heat allows remote control of cell signaling with submicron precision and unlimited penetration depth (84). As with magneto-mechanical manipulation, magneto-thermal manipulation is nondisruptive to cells, but transfection of ion channels into cells is usually needed for effective magneto-thermal manipulation. Huang et al. (85) used superparamagnetic ferrite NPs to target and control the thermosensitive transient receptor potential cation channel subfamily V member 1 (TRPV1) on the plasma membrane of neurons. Highly localized heat, which was generated in a radiofrequency magnetic field, caused a temperature increase of 0.31°C/s and triggered the activation of neurons without noticeable adverse effects. Beyond manipulation of neurons, Friedman and colleagues (86) used antibody-coated iron oxide magnetic NPs to target TRPV1 channels on the surface of HEK 293T cells. Localized heat generated by NPs activates TRPV1, which, in turn, causes the Ca^2+^-dependent synthesis of insulin in mice. In a later study, they further showed that cells can be genetically modified to synthesize iron NPs intracellularly, which were used to continuously induce insulin production under magnetic field stimulation (87). There is one crucial drawback in using antibodies to tether magnetic NPs to target specific ion channels. Because of the size of the tethering antibody, magnetic NPs are typically at a distance of 10–15 nm from the ion channels that they are tethered to. The heat generated can quickly dissipate within this physical gap. To address this issue, Chen et al. (88) showed that magnetic NPs can be used without a coating of specific ligands for the magneto-thermal stimulation of TRPV1 channels in neurons in the mouse brain. However, in this study, it is unclear why magnetic NPs were able to specifically activate TRPV1 channels without targeting ligands.

Thermosensitive ion channels can also be remotely activated by localized heat generated by plasmonic NPs when illuminated by light. Metal NPs absorb NIR light and produce localized heat (89, 90). Compared to a magnetic field, NIR does not penetrate deep tissue (91, 92) but can generate heat that is more precisely localized (91). Metal NPs, such as gold NPs, are often functionalized with ligands that target specific ion channels for highly specific and localized stimulation (91, 92). However, as discussed above in regard to magneto-thermal studies, some studies have suggested that specific targeting ligands are unnecessary. For example, Carvalho-de-Souza et al. (93) demonstrated that gold NPs functionalized with cholesterol can bind to the plasma membrane of neurons and induce the generation of membrane potentials. Because cholesterol is a general membrane component and its molecules are smaller than an antibody, its coating on gold NPs allows the NPs to bind to almost all mammalian cell types and be closer to the cell membrane for more efficient heat transfer. To improve the tissue penetration capacity of the photothermal approach, Hong and colleagues (94) developed semiconducting polymer NPs as photothermal transducers that are excited by NIR-II light (1,000–1,700 nm). Light in this wavelength window is minimally attenuated in brain tissue. As a proof of concept, NIR-II illumination, coming from a light source placed approximately 50 cm away from the head of a mouse, was shown to effectively stimulate neurons in the mouse hippocampus and motor cortex. Generally, semiconducting polymer NPs have better photothermal conversion efficiency than gold NPs (94, 95). The photothermal effect of NPs can also be used to activate ligand-gated ion channels by releasing encapsulated activator drugs. For example, Xing and colleagues (96) developed NIR-absorbing polymer NPs that, upon light illumination, release the encapsulated drug fasudil. This drug activates Kv7.4 potassium channels in cell membranes. A similar concept was also demonstrated using semiconducting polymer NPs (97) and polypyrrole NPs embedded in hydrogels (98).

In addition to the examples presented above, a variety of NPs has also been developed to control the activation of voltage-gated ion channels (99) and light-sensitive ion channels (100). A notable example is upconversion NPs, which have the unique anti-Stokes optical property of converting low-energy photons to high-energy photons. Because of this ability, they can be functionalized with specific ligands to convert NIR illumination, which penetrates tissue, to ultraviolet-visible light, which activates light-sensitive ion channels on neurons (100) and photoreceptors on cell surfaces (101).

### 3.2. Controlled Activation of Plasma Membrane Receptors

Cell signaling starts with receptor-ligand binding. For many receptors, such as G protein–coupled receptors (GPCRs) and T cell receptors, their collective clustering after ligand binding is a key step that enhances and amplifies signals from individual receptors (102-104). NPs, especially magnetic NPs, have been used to induce the clustering of receptors to control their activation. These include T cell receptors (105, 106), epidermal growth factor receptors (EGFRs) (107), Tie2 receptors (Tie2Rs) (108), DR4 receptors (109), and high-affinity immunoglobulin E (IgE) receptors (FcεRI) (110). In a typical such experiment, magnetic NPs are coated with ligands that bind to a specific receptor. As the magnetic NPs are induced to aggregate by an applied magnetic field, they cause clustering of the receptors that they bind to, triggering cell responses. In cases such as that of the immune T cell, where cell activation requires multiple stimulatory signals, magnetic NPs that are separately coated with distinct ligands can be mixed in varying ratios to tune the activation level produced in the cell (106). Interestingly, many studies have shown that the physical process of clustering is sufficient to drive receptor activation, without the need for a receptor-specific ligand. As shown by Arndt-Jovin and colleagues (107), when the ligand-binding pockets of EGFRs are blocked by antibodies, clustering of the receptors by NPs under a magnetic field can still lead to receptor phosphorylation and downstream cell signaling (Figure 2b). Lee et al. (108) also showed that magnetic NP–induced clustering of the tyrosine kinase receptor Tie2R can trigger signaling to regulate cell growth without specific ligand stimulation. Similarly, Mannix et al. (110) used monovalent ligands coated on magnetic NPs to activate Fc*ε*RI signaling in mast cells, which would otherwise be activated only by multivalent ligands.

Besides their use in magnetic control, optically responsive NPs have also been employed to control the clustering and signaling of cell-surface receptors. Liu et al. (111) immobilized, on substrates, NPs that each consisted of a gold NP core and a thermo-responsive polymer shell. After binding to specific membrane receptors and upon NIR illumination, the photothermal effect from the gold NP core caused the polymer shell to collapse and consequently pull on the cell-surface receptors. The pulling force of just a few piconewtons was sufficient to activate integrins for cell adhesion and T cell receptor signaling.

In designing NPs for controlling the clustering of receptors on cell membranes, the effects of the physiochemical parameters of the NPs, including their sizes and ligand distribution, are important considerations. For example, Huang et al. (112) showed that the size of gold NPs and the conjugation density of dinitrophenyl ligands on their surfaces control the degree of receptor cross-linking, which, in turn, either activates or inhibits downstream signaling for inflammatory responses in mast cells. Likewise, the size of NPs that interact with T cell receptors was found to significantly impact the activation level of T cells (113). Using NPs with an anisotropic coating of ligands, our group (114) has found that a partial coating of ligands on NPs activates T cells as effectively as a full coating as long as the ligand density is the same. This led to the conclusion that the conjugation density, not the total amount, of ligands on NPs is the key parameter controlling T cell receptor signaling. The spatial distribution of ligands on particles can also have a profound effect on other immune cell functions, including phagocytosis and phagosome maturation (115-117).

## 4. MANIPULATION OF INTRACELLULAR SIGNALING ACTIVITIES

Signaling pathways are intertwined in cell regulation. To understand the mechanisms governing the regulation of intracellular signaling cascades, an ideal approach is to spatially and temporally manipulate individual signaling pathways. In this section, we highlight the different NP-based strategies to control intracellular signaling through magnetic and optical manipulation (118-120).

### 4.1. Intracellular Magnetic Manipulation

**Figure 3:**
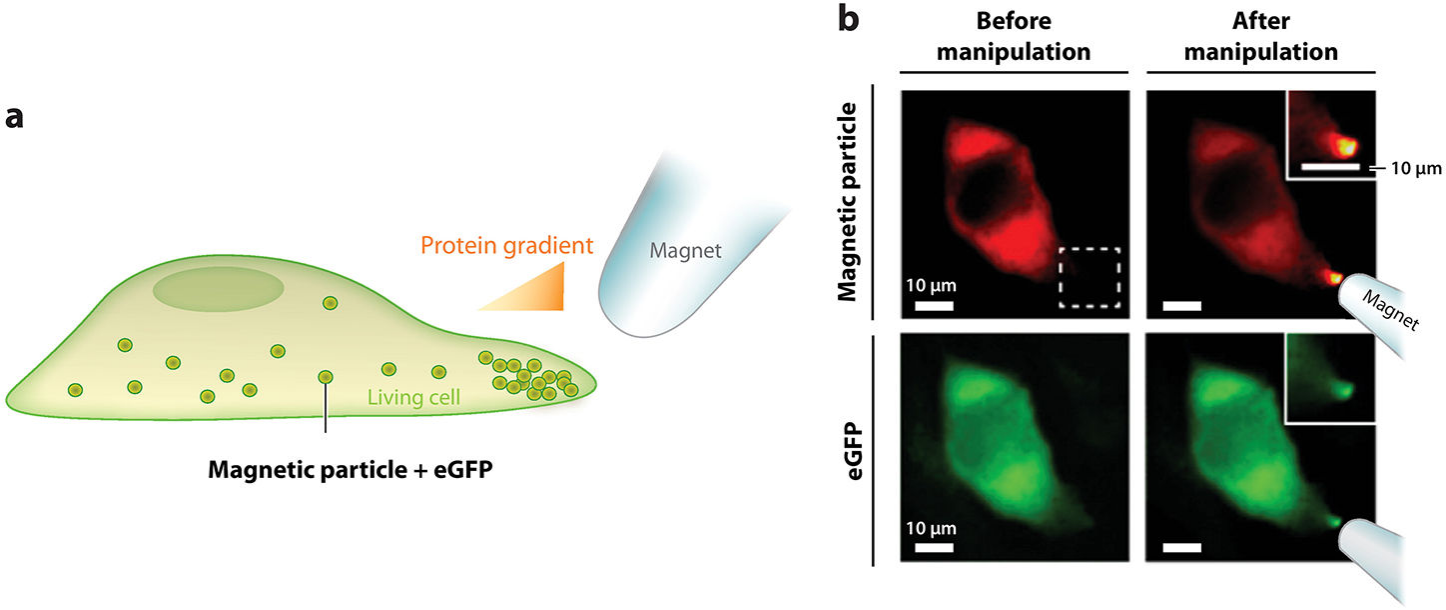
An example of magnetic manipulation of intracellular signaling activities. (*a*) Schematic showing magnetic particles conjugated with enhanced green fluorescent protein (eGFP) inside living cells. The eGFP-bound magnetic particles move and redistribute intracellularly upon the application of a magnetic field. (*b*) The magnetic field–induced redistribution of magnetic particles, conjugated with rhodamine (*red, top row*) and HaloTag ligand, generated protein gradient inside eGFP-transfected living HeLa cells (*green, bottom row*). The dotted square outlines the area from which zoomed-in images are shown as insets. Panel adapted with permission from Reference 122; copyright 2015 American Chemical Society.

Under the influence of a magnetic field, magnetic NPs aggregate and move along the force gradient. This behavior has been exploited to induce the clustering of cell-surface receptors and their downstream signaling mechanisms, as described in Section 3. Once magnetic NPs are inside cells, their aggregation in a magnetic field can be used to control the spatial distribution of signaling molecules in cells to thereby alter cell functions (Figure 3). Using magnetic NPs (120 nm), Hoffmann et al. (121) spatiotemporally controlled the assembly of microtubules in *Xenopus* egg extract, which mimics the cell cytosol, to understand signaling pathways that regulate cell morphogenesis. The magnetic NPs were coated with the protein RanGTP, a microtubule self-assembly regulatory protein. When the magnetic NPs aggregated under the influence of a magnetic field, they became hotspots of RanGTPs at a concentration high enough to trigger microtubule assembly. Thus, the distribution and orientation of microtubule fibers can be controlled by the magnitude and direction of the magnetic field. Dahan and coworkers (18) demonstrated a similar strategy in living cells by magnetically controlling Rho-GTPase signaling in the regulation of cell protrusion and migration. Ferromagnetic NPs (500 nm) that were coated with signaling proteins were microinjected into the cytoplasm of cells. The NPs then served as signaling nucleation sites that could be magnetically controlled to spatially regulate the polymerization of actin cytoskeleton and protrusion of cell membranes. Together, these studies demonstrated how the aggregation of magnetic NPs caused by a magnetic field can be used as a mechanical switch to spatiotemporally manipulate the signaling gradient. This would otherwise be challenging to do. Such techniques are powerful for dissecting the spatiotemporal regulatory function of a broad range of signaling pathways. A potential concern is that the large size of the NPs used in those studies may hinder their intracellular mobility, rendering them unsuitable for studies on some signaling activities. However, this can be optimized by choosing an appropriate combination of NP size and surface properties (122).

After being internalized into endosomes, magnetic NPs can be used to control the subcellular positioning of endosomes. Steketee et al. (123) used this approach to understand the mechanisms of neuron growth. By magnetically controlling the subcellular location of endosomes that encapsulated superparamagnetic NPs (50 nm), they revealed directly how the spatiotemporal distribution of endosomes in cells regulates various aspects of the neuron growth, including growth cone motility, protrusion dynamics, and neurite growth (123). Our group (124) recently used magnetic manipulation to control the transport velocity of phagosomes, endosome-like vacuoles that ingest pathogens, in macrophage cells. By magnetically manipulating the motility of phagosomes, we showed that the centripetal transport of phagosomes controls the kinetic rate of pathogen degradation inside them.

Magnetic NPs are also useful tools to probe the mechanical properties cells, such as the local viscoelasticity of the intracellular environment. As demonstrated by Wilhelm et al. (125), endosomes containing internalized magnetic NPs (8 nm) formed chains under the influence of a magnetic field. The viscosity and relaxation time of those chains in response to a rotating magnetic field can be used to measure the local viscoelasticity of the cell cytoplasm. A similar magnetic microrheology method was also used to measure the influence of the cytoskeleton and molecular motors on endosome transport and the viscoelasticity of the intracellular environment surrounding endosomes (126), as well as the mechanical properties of the cell nuclear interior (127).

### 4.2. Intracellular Optical Manipulation

The intracellular delivery of genetic materials is, technically, a manipulation of cell signaling. Light-controlled release of materials from NPs allows such manipulation to be activated remotely (128-132). One means of light-triggered release is through the photothermal effect that occurs in metal NPs. Sukhorukov and coworkers (133) developed multilayered polyelectrolyte microcapsules doped with silver NPs. Upon NIR illumination, the silver NPs generated localized heat that caused a polymer capsule to shrink and then release the encapsulated material. As shown by Braun et al. (134), metal NPs with optimized architecture and structure can generate sufficient heat to induce an endosome to rupture in a living cell for the delivery of small-interfering RNAs (siRNAs) in a spatiotemporally controlled manner. The photothermal effect can be harnessed to induce cell apoptosis (135, 136). Xie et al. (17) then used the NIR response of upconversion NPs to control the intracellular localization of transcriptional factors and the subsequent cell response. The NPs were coated with photocleavable DNA hybrids linked with aptamers that capture the RelA protein, a subunit of the transcriptional factor. The NP-captured RelA cannot translocate into the cell nucleus to activate a transcriptional response owing to the large size of NPs. Instead, RelA was released in the cell cytoplasm through NIR illumination to activate the transcriptional response there.

Like magnetic tweezers, optical trapping enables subcellular control of nanosized objects, from NPs and intracellular organelles to single molecules (137-139). Guo et al. (140) demonstrated the use of the optical trapping of endocytosed polystyrene NPs (500 nm) to measure the microrheology of the cytoplasm. Mallik and coworkers (141) employed optical trapping to investigate the distinct contributions from two types of molecular motors, dynein and kinesin, in endosome transport and fission. Cui and coworkers (142) used the force produced by optical trapping to detach NP-loaded endosomes from microtubules in living neurons. Because the recoil velocity of endosomes after this detachment indicates dynein forces, they revealed that a single axonal endosome can recruit up to seven dynein molecules for effective retrograde axonal transport. Because the optical trapping force originated from the refractive index mismatch between the trapped NPs and their surrounding medium (143), NPs with carefully selected refractive index must be used. However, Shan et al. (144) developed a high-lanthanide ion-doped NaYF_4_ nanocrystal for the purpose of bypassing the requirement of reflective index mismatch for optical trapping. Those nanocrystals have an improved ability to polarize under resonance conditions, an ability that consequently enhanced optical trapping forces compared to gold NPs of the same size.

## 5. SUMMARY AND PERSPECTIVES

The process of signal transduction governs how cells sense and respond to the extracellular environment. It controls not only how cells function, but also how they respond to therapeutic interventions. However, the intrinsic complexity of cell signaling is beyond what is accessible using conventional biochemical and biological assays. In this review, we highlighted how NP-based tools enable precise quantitative investigation of cell signaling–related parameters through optical imaging, as well as the direct manipulation of cell signaling processes. For quantitative measurement through imaging, NP-based optical probes are advantageous over small-molecule probes. This is because they offer better photostability, higher sensitivity, and lower cell cytotoxicity. Acting as energy transducers, NPs can convert energy from light or magnetic field to localized heat for cell signaling modulation (85, 86, 93, 94). The physical displacements of NPs under magnetic or optical control can be exploited to measure the mechanical properties of the intracellular environment (126, 127, 141, 142) or to spatiotemporally manipulate cell signaling (17, 18, 121, 134). The pioneering studies conducted to date have only scratched the surface. They indicate the remarkable untapped potential of nanotechnology-based analytical and physical tools to decipher the mechanisms of cell signal transduction in ways that would be impossible with traditional methods. However, compared with the extensive applied research on NPs for biomedical applications such as drug delivery, the use of NPs for basic research on intracellular function is still in its infancy.

There are four general challenges that must be overcome to enable the broad application of NPs in fundamental studies of cell signaling. The first general challenge is the specificity of targeting, detection, and manipulation. For targeting a specific protein or subcellular organelle, NPs are often coated with appropriately chosen ligands, which confer significantly improved specificity over small-molecule probes. While targeting plasma membrane proteins is straightforward, the targeting of NPs to intracellular locations has, so far, been mostly limited to the cell cytoplasm (by firing them into the cell ballistically) and endosomes/lysosomes (by engulfment and internalization of them by the cell through the process of endocytosis). The challenge of how to tether NP probes to other subcellular organelles remains unsolved. Regarding the specificity of detection and manipulation, a major challenge is the detection of cross talk between different species of signaling molecules or ions. For example, many fluorescent probes, such as those for Ca^2+^, are often also influenced by pH (145). This confounding of influences could be overcome by developing highly specific fluorescent probes or novel NP designs. One example of such specificity is that attained by using DNAzymes that are responsive to only one type of ion.

The second general challenge is to improve the capacity for multiplexing detection and manipulation. This will be critical for disentangling the relationships between different signaling molecules or pathways. A few types of multifunctional NP probes have been developed, but studies have heavily focused on the synthesis, providing very few insights into cell biology.

The third general challenge is to increase the spatiotemporal resolution of the imaging and manipulation of cell signaling processes. Because cells respond to even subtle gradients of signaling molecules, it would be ideal to enable the detection of single analytes or manipulate the intracellular positioning of single molecules with temporal resolutions that match those of the physiological fluctuations.

Lastly, there is the general challenge of making NP approaches widely accessible to biology laboratories to enable the broad application of the new techniques to important biology problems.
